# Supplementary figures and images for: Structure-guided design of VAR2CSA-based immunogens and a cocktail strategy for a placental malaria vaccine
Source: PLoS Pathog. 2024 Mar 4;20(3):e1011879. doi: 10.1371/journal.ppat.1011879 (PMC10939253; doi:10.1371/journal.ppat.1011879)

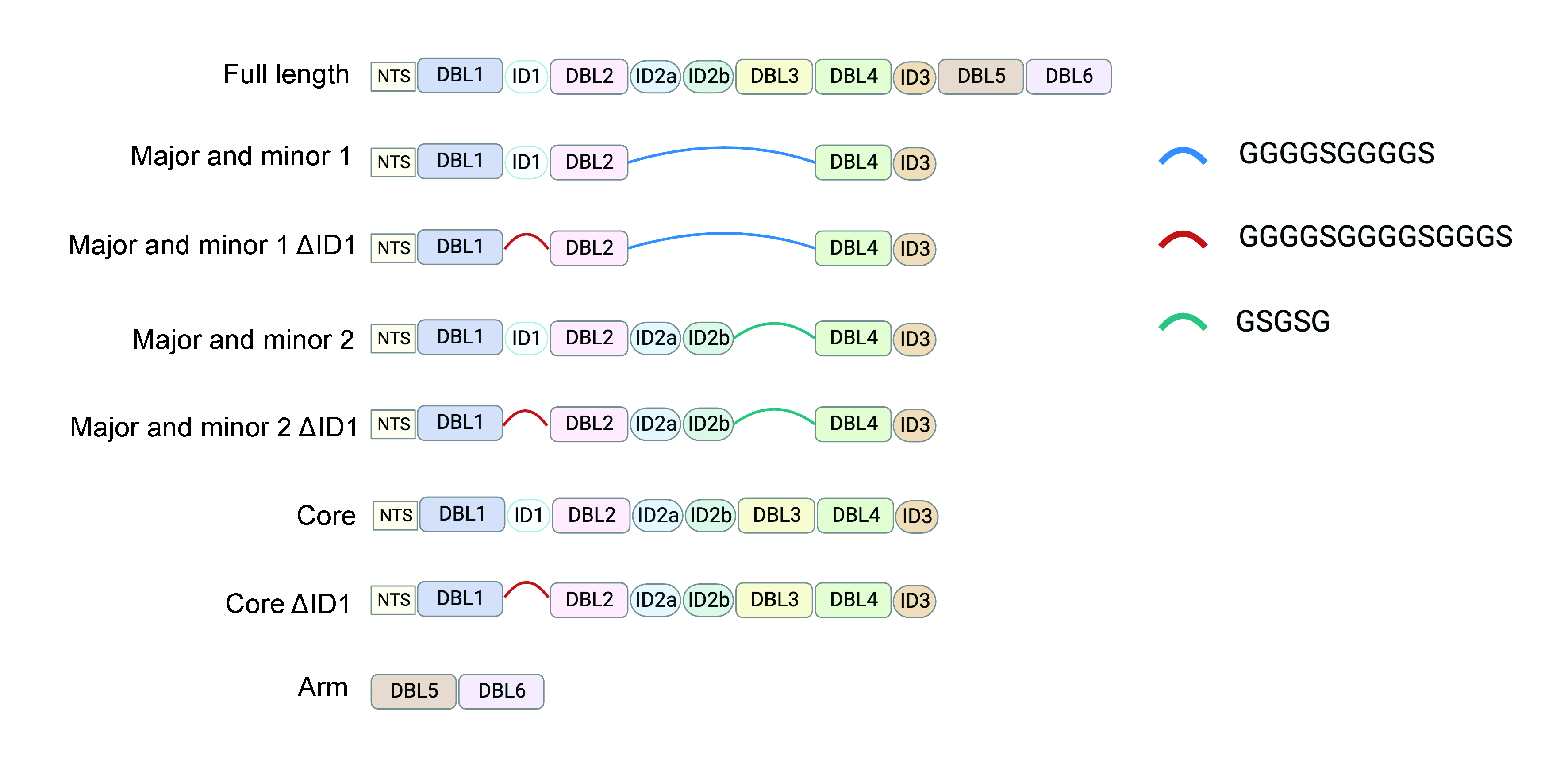

Supplement: S1 Fig — Each immunogen is illustrated as a schematic representation and the sequences of the linker was shown. This figure was generated with the help of Biorender (https://www.biorender.com/) and PRISM 9. (TIFF) [file ppat.1011879.s001.tiff]

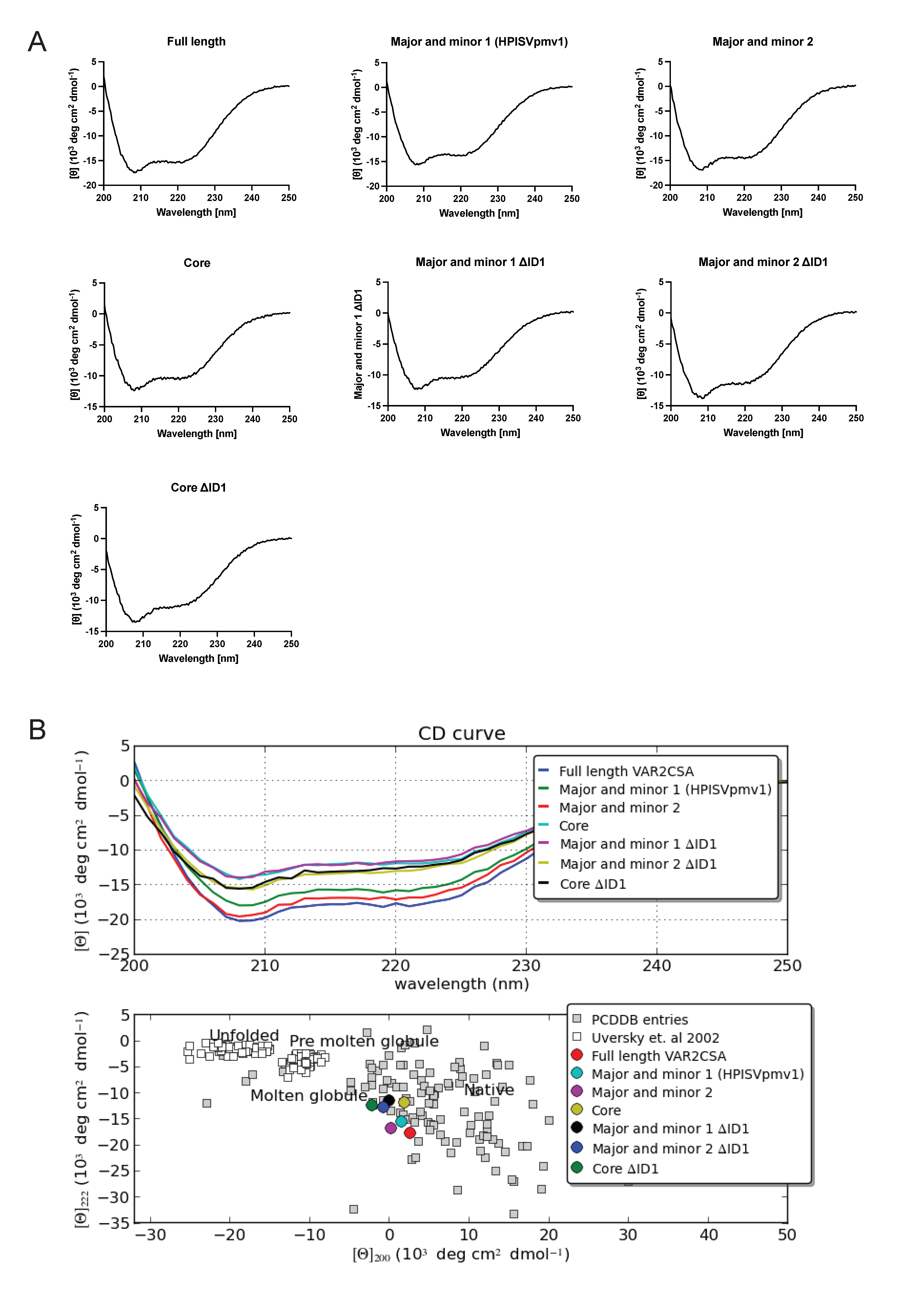

Supplement: S3 Fig — (A) CD spectra of the immunogens. (B) Upper panel shows the CD spectra for the different immunogens as color-coded in the inset. The lower panel shows the MRE (Mean-residue-ellipticity) values at lambda = 222 nm versus lambda = 200 nm that indicate the folding state of each immunogen as color-coded in the inset. PCDDB—Protein Circular Dichroism Data Bank. (TIFF) [file ppat.1011879.s003.tiff]

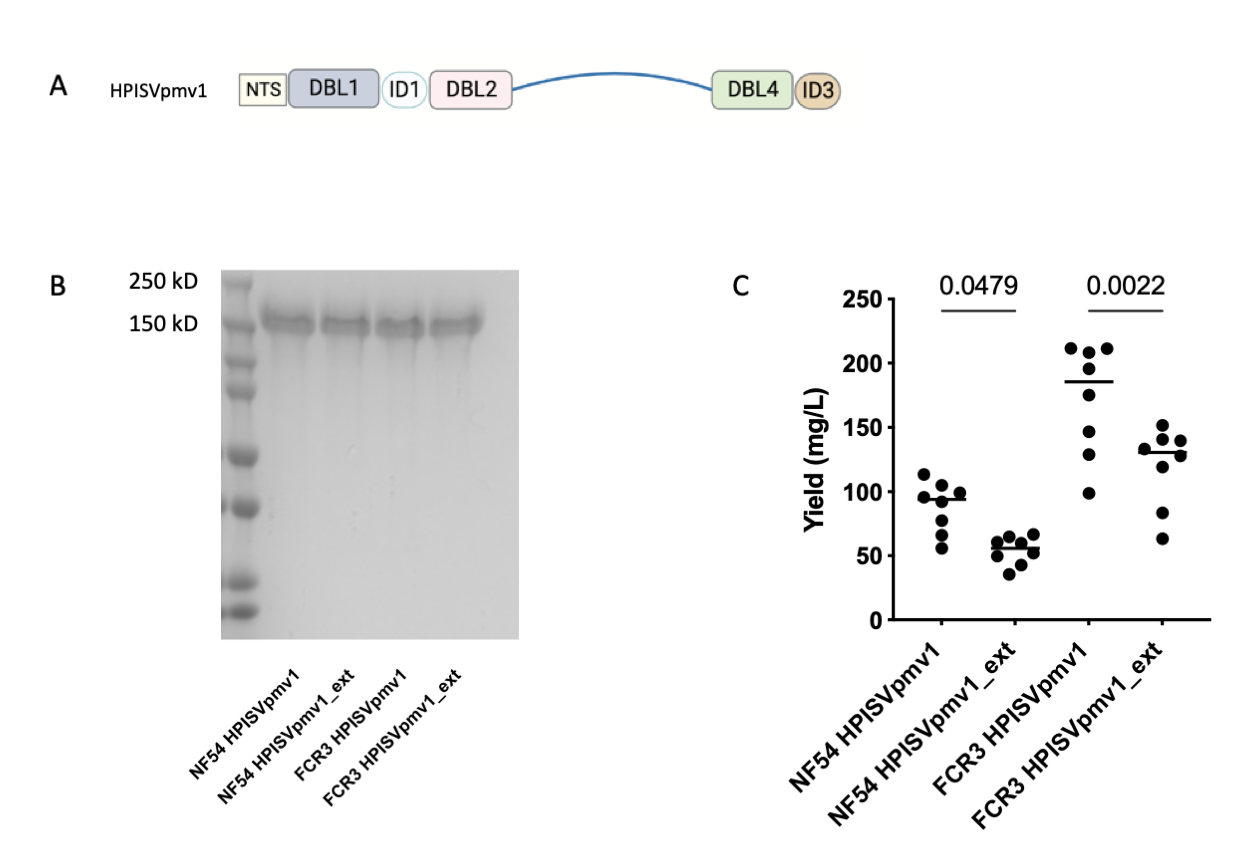

Supplement: S4 Fig — (A) Domain structure of the HPISVpmv1 domain structure. (B) SDS-PAGE analysis result of HPISVpmv1 and HPISVpmv1_ext from NF54 and FCR3 strains. ‘ext’ is short for extension. (C) The yield of each immunogen is determined using eight biological replicates. The p values of the one-way ANOVA test are shown. This figure was generated with the help of Biorender (https://www.biorender.com/). (TIFF) [file ppat.1011879.s004.tiff]

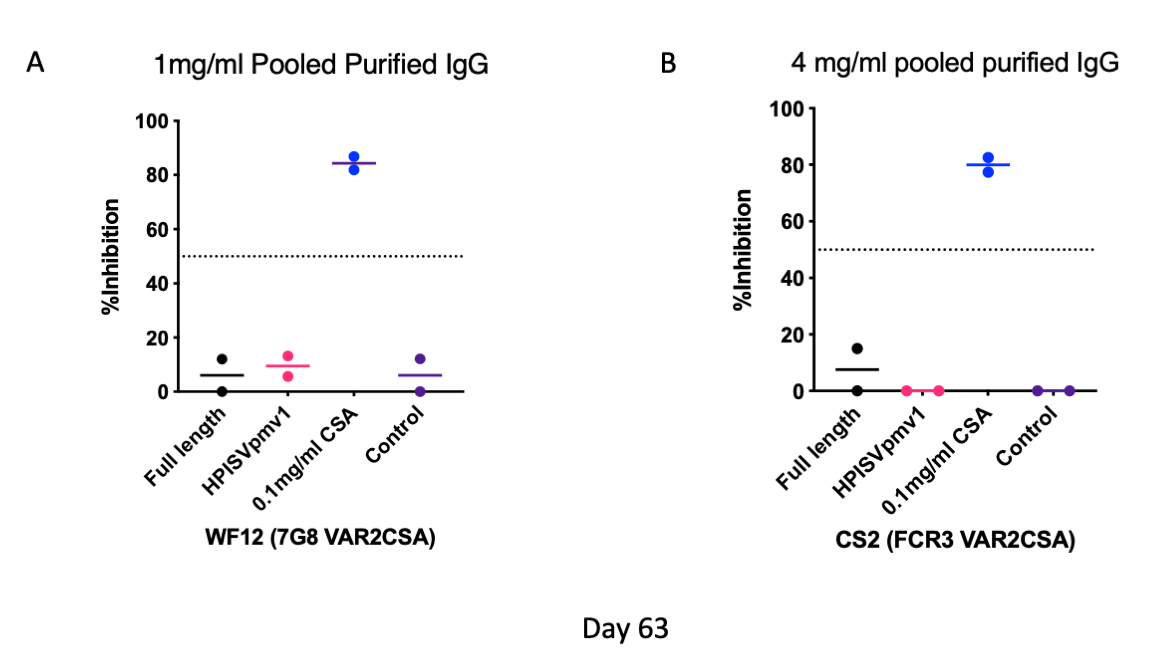

Supplement: S5 Fig — (A) The BIA assay results against WF12 parasite strain containing 7G8 VAR2CSA using purified pooled IgG at 1mg/ml from the serum after three vaccinations. (B) The BIA assay results against CS2 parasite strain containing FCR3 VAR2CSA using purified pooled IgG at 4mg/ml from the serum after three vaccinations. The dashed line indicates the 50% inhibition level used as a cutoff for inhibitory or non-inhibitory activity, the data shown are derived from two independent experiments and each point is the mean of two technical replicates. (TIFF) [file ppat.1011879.s005.tiff]

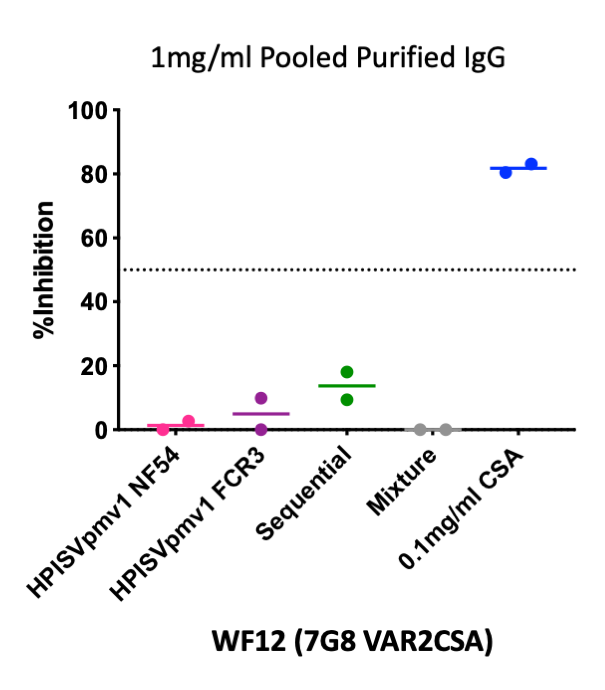

Supplement: S6 Fig — The BIA assay results against WF12 parasite strain containing 7G8 VAR2CSA using purified pooled IgG at 1mg/ml from the serum after two vaccinations. The dashed line indicates the 50% inhibition level used as a cutoff for inhibitory or non-inhibitory activity, the data shown are derived from two independent experiments and each point is the mean of two technical replicates. (TIFF) [file ppat.1011879.s006.tiff]

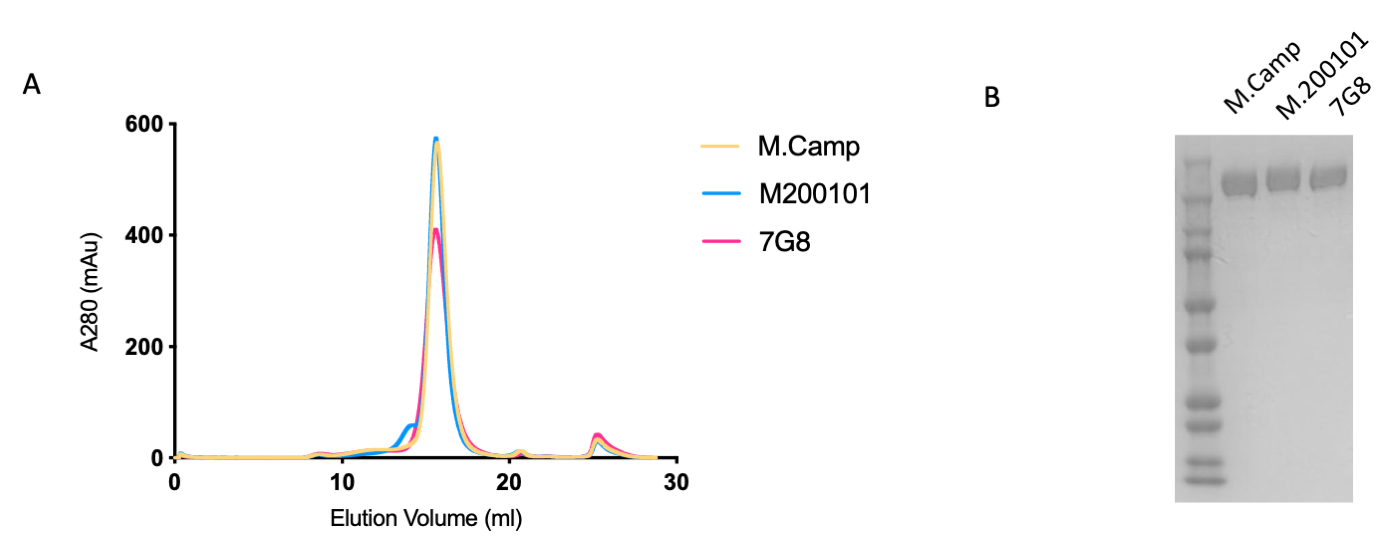

Supplement: S7 Fig — The Size-exclusion chromatography (SEC) profile (A) and SDS PAGE gel (B) of HPISVpmv1 from 7G8, M.Camp and M200101 are shown. (TIFF) [file ppat.1011879.s007.tiff]
