# Supplementary material for: Structure-guided design of VAR2CSA-based immunogens and a cocktail strategy for a placental malaria vaccine
Source: PLoS Pathog. 2024 Mar 4;20(3):e1011879. doi: 10.1371/journal.ppat.1011879 (PMC10939253; doi:10.1371/journal.ppat.1011879)
Supplement: S1 Table — The sequence identity (%) between each of the two HPISVpmv1 strains are shown in the table. (DOCX) [file ppat.1011879.s008.docx]

|  | NF54 | FCR3 | M.Camp | M200101 |
| --- | --- | --- | --- | --- |
| FCR3 | 81.85 |  |  |  |
| M.Camp | 86.62 | 79.42 |  |  |
| M200101 | 78.37 | 81.23 | 78.33 |  |
| 7G8 | 82.22 | 81.74 | 77.95 | 82.45 |

Table S1. Sequence identity among 5 HPISVpmv1 strains. The sequence identity (%) between each of the two HPISVpmv1 strains are shown in the table.
