# Supplementary material for: Structure-guided design of VAR2CSA-based immunogens and a cocktail strategy for a placental malaria vaccine
Source: PLoS Pathog. 2024 Mar 4;20(3):e1011879. doi: 10.1371/journal.ppat.1011879 (PMC10939253; doi:10.1371/journal.ppat.1011879)
Supplement: S2 Table — (DOCX) [file ppat.1011879.s009.docx]

Table S2. Protein sequences of the designed immunogens.

| VAR2CSA NF54 CORE | MDKSSIANKIEAYLGAKSDDSKIDQSLKADPSEVQYYGSGGDGYYLRKNICKITVNHSDSGTNDPCDRIPPPYGDNDQWKCAIILSKVSEKPENVFVPPRRQRMCINNLEKLNVDKIRDKHAFLADVLLTARNEGERIVQNHPDTNSSNVCNALERSFADIADIIRGTDLWKGTNSNLEQNLKQMFAKIRENDKVLQDKYPKDQNYRKLREDWWNANRQKVWEVITCGARSNDLLIKRGWRTSGKSNGDNKLELCRKCGHYEEKVPTKLDYVPQFLRWLTEWIEDFYREKQNLIDDMERHREECTSEDHKSKEGTSYCSTCKDKCKKYCECVKKWKSEWENQKNKYTELYQQNKNETSQKNTSRYDDYVKDFFKKLEANYSSLENYIKGDPYFAEYATKLSFILNSSDANNPSEKIQKNNDEVCNCNESGIASVEQEQISDPSSNKTCITHSSIKANKKKVCKHVKLGVRENDKDLRVCVIEHTSLSGVENCCCQDFLRILQENCSDNKSGSSSNGSCNNKNQEACEKNLEKVLASLTNCYKCDKCKSEQSKKNNKNWIWKKSSGKEGGLQKEYANTIGLPPRTQSLCLVVCLDEKGKKTQELKNIRTNSELLKEWIIAAFHEGKNLKPSHEKKNDDNGKKLCKALEYSFADYGDLIKGTSIWDNEYTKDLELNLQKIFGKLFRKYIKKNNTAEQDTSYSSLDELRESWWNTNKKYIWLAMKHGAGMNSTTCCGDGSVTGSGSSCDDIPTIDLIPQYLRFLQEWVEHFCKQRQEKVKPVIENCKSCKESGGTCNGECKTECKNKCEVYKKFIEDCKGGDGTAGSSWVKRWDQIYKRYSKYIEDAKRNRKAGTKNCGPSSTTNAAENKCVQSDIDSFFKHLIDIGLTTPSSYLSIVLDDNICGADKAPWTTYTTYTTTEKCNKETDKSKLQQCNTAVVVNVPSPLGNTPHGYKYACQCKIPTNEETCDDRKEYMNQWSCGSARTMKRGYKNDNYELCKYNGVDVKPTTVRSNSSKLDDKDVTFFNLFEQWNKEIQYQIEQYMTNTKISCNNEKNVLSRVSDEAAQPKFSDNERDRNSITHEDKNCKEKCKCYSLWIEKINDQWDKQKDNYNKFQRKQIYDANKGSQNKKVVSLSNFLFFSCWEEYIQKYFNGDWSKIKNIGSDTFEFLIKKCGNDSGDGETIFSEKLNNAEKKCKENESTNNKMKSSETSCDCSEPIYIRGCQPKIYDGKIFPGKGGEKQWICKDTIIHGDTNGACIPPRTQNLCVGELWDKRYGGRSNIKNDTKESLKQKIKNAIQKETELLYEYHDKGTAIISRNPMKGQKEKEEKNNDSNGLPKGFCHAVQRSFIDYKNMILGTSVNIYEYIGKLQEDIKKIIEKGTTKQNGKTVGSGAENVNAWWKGIEGEMWDAVRCAITKINKKQKKNGTFSIDECGIFPPTGNDEDQSVSWFKEWSEQFCIERLQYEKNIRDACTNNGQGDKIQGDCKRKCEEYKKYISEKKQEWDKQKTKYENKYVGKSASDLLKENYPECISANFDFIFNDNIEYKTYYPYGDYSSICSCEQVKYYEYNNAEKKNNKSLCHEKGNDRTWSKKYIKKLENGRTLEGVYVPPRRQQLCLYELFPIIIKNKNDITNAKKELLETLQIVAEREAYYLWKQYHAHNDTTYLAHKKACCAIRGSFYDLEDIIKGNDLVHDEYTKYIDSKLNEIFDSSNKNDIETKRARTDWWENEAIAVPNITGANKSDPKTIRQLVWDAMQSGVRKAIDEEKEKKKPNENFPPCMGVQHIGIAKPQFIRWLEEWTNEFCEKYTKYFEDMKSNCNLRKGADDCDDNSNIECKKACANYTNWLNPKRIEWNGMSNYYNKIYRKSNKESEDGKDYSMIMEPTVIDYLNKRCNGEINGNYICCSCKNIGENSTSGTVNKKLQKKETQCEDNKGPLDLMNKVLNKMDPKYSEHKM |
| --- | --- |
| VAR2CSA NF54 Major and minor 1 (HPISVpmv1 NF54) | MDKSSIANKIEAYLGAKSDDSKIDQSLKADPSEVQYYGSGGDGYYLRKNICKITVNHSDSGTNDPCDRIPPPYGDNDQWKCAIILSKVSEKPENVFVPPRRQRMCINNLEKLNVDKIRDKHAFLADVLLTARNEGERIVQNHPDTNSSNVCNALERSFADIADIIRGTDLWKGTNSNLEQNLKQMFAKIRENDKVLQDKYPKDQNYRKLREDWWNANRQKVWEVITCGARSNDLLIKRGWRTSGKSNGDNKLELCRKCGHYEEKVPTKLDYVPQFLRWLTEWIEDFYREKQNLIDDMERHREECTSEDHKSKEGTSYCSTCKDKCKKYCECVKKWKSEWENQKNKYTELYQQNKNETSQKNTSRYDDYVKDFFKKLEANYSSLENYIKGDPYFAEYATKLSFILNSSDANNPSEKIQKNNDEVCNCNESGIASVEQEQISDPSSNKTCITHSSIKANKKKVCKHVKLGVRENDKDLRVCVIEHTSLSGVENCCCQDFLRILQENCSDNKSGSSSNGSCNNKNQEACEKNLEKVLASLTNCYKCDKCKSEQSKKNNKNWIWKKSSGKEGGLQKEYANTIGLPPRTQSLCLVVCLDEKGKKTQELKNIRTNSELLKEWIIAAFHEGKNLKPSHEKKNDDNGKKLCKALEYSFADYGDLIKGTSIWDNEYTKDLELNLQKIFGKLFRKYIKKNNTAEQDTSYSSLDELRESWWNTNKKYIWLAMKHGAGMNSTTCCGDGSVTGSGSSCDDIPTIDLIPQYLRFLQEWVEHFCKQRQEKVKPVIENCKSCKESGGTCNGECKTECKNKCEVYKKFIEDCKGGDGTAGSSWVKRWDQIYKRYSKYIEDAKRNRKAGTKNCGPSSTTNAAENKCVQSDIDSFFKHLIDIGLTTPSSYLSIVLDDNICGADKAPWTTYTTYTTTEKCNKETDKSKLQQCNTAVVVNVPSPLGNTPHGYKYACQCKIPGGGGSGGGGSAEKKNNKSLCHEKGNDRTWSKKYIKKLENGRTLEGVYVPPRRQQLCLYELFPIIIKNKNDITNAKKELLETLQIVAEREAYYLWKQYHAHNDTTYLAHKKACCAIRGSFYDLEDIIKGNDLVHDEYTKYIDSKLNEIFDSSNKNDIETKRARTDWWENEAIAVPNITGANKSDPKTIRQLVWDAMQSGVRKAIDEEKEKKKPNENFPPCMGVQHIGIAKPQFIRWLEEWTNEFCEKYTKYFEDMKSNCNLRKGADDCDDNSNIECKKACANYTNWLNPKRIEWNGMSNYYNKIYRKSNKESEDGKDYSMIMEPTVIDYLNKRCNGEINGNYICCSCKNIGENSTSGTVNKKLQKKETQCEDNKGPLDLMNKVLNKMDPKYSEHKM |
| VAR2CSA NF54 Major and minor 2 | MDKSSIANKIEAYLGAKSDDSKIDQSLKADPSEVQYYGSGGDGYYLRKNICKITVNHSDSGTNDPCDRIPPPYGDNDQWKCAIILSKVSEKPENVFVPPRRQRMCINNLEKLNVDKIRDKHAFLADVLLTARNEGERIVQNHPDTNSSNVCNALERSFADIADIIRGTDLWKGTNSNLEQNLKQMFAKIRENDKVLQDKYPKDQNYRKLREDWWNANRQKVWEVITCGARSNDLLIKRGWRTSGKSNGDNKLELCRKCGHYEEKVPTKLDYVPQFLRWLTEWIEDFYREKQNLIDDMERHREECTSEDHKSKEGTSYCSTCKDKCKKYCECVKKWKSEWENQKNKYTELYQQNKNETSQKNTSRYDDYVKDFFKKLEANYSSLENYIKGDPYFAEYATKLSFILNSSDANNPSEKIQKNNDEVCNCNESGIASVEQEQISDPSSNKTCITHSSIKANKKKVCKHVKLGVRENDKDLRVCVIEHTSLSGVENCCCQDFLRILQENCSDNKSGSSSNGSCNNKNQEACEKNLEKVLASLTNCYKCDKCKSEQSKKNNKNWIWKKSSGKEGGLQKEYANTIGLPPRTQSLCLVVCLDEKGKKTQELKNIRTNSELLKEWIIAAFHEGKNLKPSHEKKNDDNGKKLCKALEYSFADYGDLIKGTSIWDNEYTKDLELNLQKIFGKLFRKYIKKNNTAEQDTSYSSLDELRESWWNTNKKYIWLAMKHGAGMNSTTCCGDGSVTGSGSSCDDIPTIDLIPQYLRFLQEWVEHFCKQRQEKVKPVIENCKSCKESGGTCNGECKTECKNKCEVYKKFIEDCKGGDGTAGSSWVKRWDQIYKRYSKYIEDAKRNRKAGTKNCGPSSTTNAAENKCVQSDIDSFFKHLIDIGLTTPSSYLSIVLDDNICGADKAPWTTYTTYTTTEKCNKETDKSKLQQCNTAVVVNVPSPLGNTPHGYKYACQCKIPTNEETCDDRKEYMNQWSCGSARTMKRGYKNDNYELCKYNGVDVKPTTVRSNSSKLDDKDVTFFNLFEQWNKEIQYQIEQYMTNTKISCNNEKNVLSRVSDEAAQPKFSDNERDRNSITHEDKNCKEKCKCYSLWIEKINDQWDKQKDNYNKFQRKQIYDANKGSQNKKVVSLSNFLFFSCWEEYIQKYFNGDWSKIKNIGSDTFEFLIKKCGNDSGDGETIFSEKLNNAEKKCKENESTNNKMKSSETSGSGSGAEKKNNKSLCHEKGNDRTWSKKYIKKLENGRTLEGVYVPPRRQQLCLYELFPIIIKNKNDITNAKKELLETLQIVAEREAYYLWKQYHAHNDTTYLAHKKACCAIRGSFYDLEDIIKGNDLVHDEYTKYIDSKLNEIFDSSNKNDIETKRARTDWWENEAIAVPNITGANKSDPKTIRQLVWDAMQSGVRKAIDEEKEKKKPNENFPPCMGVQHIGIAKPQFIRWLEEWTNEFCEKYTKYFEDMKSNCNLRKGADDCDDNSNIECKKACANYTNWLNPKRIEWNGMSNYYNKIYRKSNKESEDGKDYSMIMEPTVIDYLNKRCNGEINGNYICCSCKNIGENSTSGTVNKKLQKKETQCEDNKGPLDLMNKVLNKMDPKYSEHKM |
| VAR2CSA NF54 CORE ΔID1 | MDKSSIANKIEAYLGAKSDDSKIDQSLKADPSEVQYYGSGGDGYYLRKNICKITVNHSDSGTNDPCDRIPPPYGDNDQWKCAIILSKVSEKPENVFVPPRRQRMCINNLEKLNVDKIRDKHAFLADVLLTARNEGERIVQNHPDTNSSNVCNALERSFADIADIIRGTDLWKGTNSNLEQNLKQMFAKIRENDKVLQDKYPKDQNYRKLREDWWNANRQKVWEVITCGARSNDLLIKRGWRTSGKSNGDNKLELCRKCGHYEEKVPTKLDYVPQFLRWLTEWIEDFYREKQNLIDDMERHREECTSEDHKSKEGTSYCSTCKDKCKKYCECVKKWKSEWENQKNKYTELYQQNKNETSQKNTSRYDDYVKDFFKKLEANYSSLENYIKGGGGSGGGGSGGGSKSEQSKKNNKNWIWKKSSGKEGGLQKEYANTIGLPPRTQSLCLVVCLDEKGKKTQELKNIRTNSELLKEWIIAAFHEGKNLKPSHEKKNDDNGKKLCKALEYSFADYGDLIKGTSIWDNEYTKDLELNLQKIFGKLFRKYIKKNNTAEQDTSYSSLDELRESWWNTNKKYIWLAMKHGAGMNSTTCCGDGSVTGSGSSCDDIPTIDLIPQYLRFLQEWVEHFCKQRQEKVKPVIENCKSCKESGGTCNGECKTECKNKCEVYKKFIEDCKGGDGTAGSSWVKRWDQIYKRYSKYIEDAKRNRKAGTKNCGPSSTTNAAENKCVQSDIDSFFKHLIDIGLTTPSSYLSIVLDDNICGADKAPWTTYTTYTTTEKCNKETDKSKLQQCNTAVVVNVPSPLGNTPHGYKYACQCKIPTNEETCDDRKEYMNQWSCGSARTMKRGYKNDNYELCKYNGVDVKPTTVRSNSSKLDDKDVTFFNLFEQWNKEIQYQIEQYMTNTKISCNNEKNVLSRVSDEAAQPKFSDNERDRNSITHEDKNCKEKCKCYSLWIEKINDQWDKQKDNYNKFQRKQIYDANKGSQNKKVVSLSNFLFFSCWEEYIQKYFNGDWSKIKNIGSDTFEFLIKKCGNDSGDGETIFSEKLNNAEKKCKENESTNNKMKSSETSCDCSEPIYIRGCQPKIYDGKIFPGKGGEKQWICKDTIIHGDTNGACIPPRTQNLCVGELWDKRYGGRSNIKNDTKESLKQKIKNAIQKETELLYEYHDKGTAIISRNPMKGQKEKEEKNNDSNGLPKGFCHAVQRSFIDYKNMILGTSVNIYEYIGKLQEDIKKIIEKGTTKQNGKTVGSGAENVNAWWKGIEGEMWDAVRCAITKINKKQKKNGTFSIDECGIFPPTGNDEDQSVSWFKEWSEQFCIERLQYEKNIRDACTNNGQGDKIQGDCKRKCEEYKKYISEKKQEWDKQKTKYENKYVGKSASDLLKENYPECISANFDFIFNDNIEYKTYYPYGDYSSICSCEQVKYYEYNNAEKKNNKSLCHEKGNDRTWSKKYIKKLENGRTLEGVYVPPRRQQLCLYELFPIIIKNKNDITNAKKELLETLQIVAEREAYYLWKQYHAHNDTTYLAHKKACCAIRGSFYDLEDIIKGNDLVHDEYTKYIDSKLNEIFDSSNKNDIETKRARTDWWENEAIAVPNITGANKSDPKTIRQLVWDAMQSGVRKAIDEEKEKKKPNENFPPCMGVQHIGIAKPQFIRWLEEWTNEFCEKYTKYFEDMKSNCNLRKGADDCDDNSNIECKKACANYTNWLNPKRIEWNGMSNYYNKIYRKSNKESEDGKDYSMIMEPTVIDYLNKRCNGEINGNYICCSCKNIGENSTSGTVNKKLQKKETQCEDNKGPLDLMNKVLNKMDPKYSEHKM |
| VAR2CSA NF54 Major and minor 1 ΔID1 | MDKSSIANKIEAYLGAKSDDSKIDQSLKADPSEVQYYGSGGDGYYLRKNICKITVNHSDSGTNDPCDRIPPPYGDNDQWKCAIILSKVSEKPENVFVPPRRQRMCINNLEKLNVDKIRDKHAFLADVLLTARNEGERIVQNHPDTNSSNVCNALERSFADIADIIRGTDLWKGTNSNLEQNLKQMFAKIRENDKVLQDKYPKDQNYRKLREDWWNANRQKVWEVITCGARSNDGSGSGLELCRKCGHYEEKVPTKLDYVPQFLRWLTEWIEDFYREKQNLIDDMERHREECTSEDHKSKEGTSYCSTCKDKCKKYCECVKKWKSEWENQKNKYTELYQQNKNETSQKNTSRYDDYVKDFFKKLEANYSSLENYIKGGGGSGGGGSGGGSKSEQSKKNNKNWIWKKSSGKEGGLQKEYANTIGLPPRTQSLCLVVCLDEKGKKTQELKNIRTNSELLKEWIIAAFHEGKNLKPSHEKKNDDNGKKLCKALEYSFADYGDLIKGTSIWDNEYTKDLELNLQKIFGKLFGGGGSSSLDELRESWWNTNKKYIWLAMKHGAGMNSTTCCGDGSVTGSGSSCDDIPTIDLIPQYLRFLQEWVEHFCKQRQEKVKPVIENCKSCKESGGTCNGECKTECKNKCEVYKKFIEDCKGGDGTAGSSWVKRWDQIYKRYSKYIEDAKRNRKAGTKNCGSGSGCVQSDIDSFFKHLIDIGLTTPSSYLSIVLDDNICGADKAPWTTYTTYTTTEKCNKETDKSKLQQCNTAVVVNVPSPLGNTPHGYKYACQCKIPGGGGSGGGGSGGSGSGAEKKNNKSLCHEKGNDRTWSKKYIKKLENGRTLEGVYVPPRRQQLCLYELFPIIIKNKNDITNAKKELLETLQIVAEREAYYLWKQYHAHNDTTYLAHKKACCAIRGSFYDLEDIIKGNDLVHDEYTKYIDSKLNEIFDSSNKNDIETKRARTDWWENEAIAVPNITGANKSDPKTIRQLVWDAMQSGVRKAIDEEKEKKKPNENFPPCMGVQHIGIAKPQFIRWLEEWTNEFCEKYTKYFEDMKSNCNLRKGADDCDDNSNIECKKACANYTNWLNPKRIEWNGMSNYYNKIYRKSNKESEDGKDYSMIMEPTVIDYLNKRCNGEINGNYICCSCKNIGENSTSGTVNKKLQKKETQCEDNKGPLDLMNKVLNKMDPKYSEHKM |
| VAR2CSA NF54 Major and minor 2 ΔID1 | MDKSSIANKIEAYLGAKSDDSKIDQSLKADPSEVQYYGSGGDGYYLRKNICKITVNHSDSGTNDPCDRIPPPYGDNDQWKCAIILSKVSEKPENVFVPPRRQRMCINNLEKLNVDKIRDKHAFLADVLLTARNEGERIVQNHPDTNSSNVCNALERSFADIADIIRGTDLWKGTNSNLEQNLKQMFAKIRENDKVLQDKYPKDQNYRKLREDWWNANRQKVWEVITCGARSNDLLIKRGWRTSGKSNGDNKLELCRKCGHYEEKVPTKLDYVPQFLRWLTEWIEDFYREKQNLIDDMERHREECTSEDHKSKEGTSYCSTCKDKCKKYCECVKKWKSEWENQKNKYTELYQQNKNETSQKNTSRYDDYVKDFFKKLEANYSSLENYIKGGGGSGGGGSGGGSKSEQSKKNNKNWIWKKSSGKEGGLQKEYANTIGLPPRTQSLCLVVCLDEKGKKTQELKNIRTNSELLKEWIIAAFHEGKNLKPSHEKKNDDNGKKLCKALEYSFADYGDLIKGTSIWDNEYTKDLELNLQKIFGKLFRKYIKKNNTAEQDTSYSSLDELRESWWNTNKKYIWLAMKHGAGMNSTTCCGDGSVTGSGSSCDDIPTIDLIPQYLRFLQEWVEHFCKQRQEKVKPVIENCKSCKESGGTCNGECKTECKNKCEVYKKFIEDCKGGDGTAGSSWVKRWDQIYKRYSKYIEDAKRNRKAGTKNCGPSSTTNAAENKCVQSDIDSFFKHLIDIGLTTPSSYLSIVLDDNICGADKAPWTTYTTYTTTEKCNKETDKSKLQQCNTAVVVNVPSPLGNTPHGYKYACQCKIPTNEETCDDRKEYMNQWSCGSARTMKRGYKNDNYELCKYNGVDVKPTTVRSNSSKLDDKDVTFFNLFEQWNKEIQYQIEQYMTNTKISCNNEKNVLSRVSDEAAQPKFSDNERDRNSITHEDKNCKEKCKCYSLWIEKINDQWDKQKDNYNKFQRKQIYDANKGSQNKKVVSLSNFLFFSCWEEYIQKYFNGDWSKIKNIGSDTFEFLIKKCGNDSGDGETIFSEKLNNAEKKCKENESTNNKMKSSETSGSGSGAEKKNNKSLCHEKGNDRTWSKKYIKKLENGRTLEGVYVPPRRQQLCLYELFPIIIKNKNDITNAKKELLETLQIVAEREAYYLWKQYHAHNDTTYLAHKKACCAIRGSFYDLEDIIKGNDLVHDEYTKYIDSKLNEIFDSSNKNDIETKRARTDWWENEAIAVPNITGANKSDPKTIRQLVWDAMQSGVRKAIDEEKEKKKPNENFPPCMGVQHIGIAKPQFIRWLEEWTNEFCEKYTKYFEDMKSNCNLRKGADDCDDNSNIECKKACANYTNWLNPKRIEWNGMSNYYNKIYRKSNKESEDGKDYSMIMEPTVIDYLNKRCNGEINGNYICCSCKNIGENSTSGTVNKKLQKKETQCEDNKGPLDLMNKVLNKMDPKYSEHKM |
| VAR2CSA NF54 Arm | KMKCTEVYLEHVEEQLKEIDNAIKDYKLYPLDRCFDDKSKMKVCDLIGDAIGCKHKTKLDELDEWNDVDMRDPYNKYKGVLIPPRRRQLCFSRIVRGPANLRNLKEFKEEILKGAQSEGKFLGNYYNEDKDKEKALEAMKNSFYDYEYIIKGSDMLTNIQFKDIKRKLDRLLEKETNNTEKVDDWWETNKKSIWNAMLCGYKKSGNKIIDPSWCTIPTTETPPQFLRWIKEWGTNVCIQKEEHKEYVKSKCSNVTNLGAQESESKNCTSEIKKYQEWSRKRSIQWEAISEGYKKYKGMDEFKNTFKNIKEPDANEPNANEYLKKHCSKCPCGFNDMQEITKYTNIGNEAFKQIKEQVDIPAELEDVIYRLKHHEYDKGNDYICNKYKNINVNMKKNNDDTWTDLVKNSSDINKGVLLPPRRKNLFLKIDESDICKYKRDPKLFKDFIYSSAISEVERLKKVYGEAKTKVVHAMKYSFADIGSIIKGDDMMENNSSDKIGKILGDGVGQNEKRKKWWDMNKYHIWESMLCGYKHAYGNISENDRKMLDIPNNDDEHQFLRWFQEWTENFCTKRNELYENMVTACNSAKCNTSNGSVDKKECTEACKNYSNFILIKKKEYQSLNSQYDMNYKETKAEKKESPEYFKDKCNGECSCLSEYFKDETRWKNPYETLDDTEVKNNCMCKPPPPASNNTSDILQKTIPF |
| HPISVpmv1 FCR3 | MDSTSTIANKIEEYLGAKSDDSKIDELLKADPSEVEYYRSGGDGDYLKNNICKITVNHSDSGKYDPCEKKLPPYDDNDQWKCQQNSSDGSGKPENICVPPRRERLCTYNLENLKFDKIRDNNAFLADVLLTARNEGEKIVQNHPDTNSSNVCNALERSFADLADIIRGTDQWKGTNSNLEKNLKQMFAKIRENDKVLQDKYPKDQKYTKLREAWWNANRQKVWEVITCGARSNDLLIKRGWRTSGKSDRKKNFELCRKCGHYEKEVPTKLDYVPQFLRWLTEWIEDFYREKQNLIDDMERHREECTREDHKSKEGTSYCSTCKDKCKKYCECVKKWKTEWENQENKYKDLYEQNKNKTSQKNTSRYDDYVKDFFEKLEANYSSLENYIKGDPYFAEYATKLSFILNPSDANNPSGETANHNDEACNCNESGISSVGQAQTSGPSSNKTCITHSSIKTNKKKECKDVKLGVRENDKDLKICVIEDTSLSGVDNCCCQDLLGILQENCSDNKRGSSSNDSCDNKNQDECQKKLEKVFASLTNGYKCDKCKSGTSRSKKKWIWKKSSGNEEGLQEEYANTIGLPPRTQSLYLGNLPKLENVCEDVKDINFDTKEKFLAGCLIVSFHEGKNLKKRYPQNKNSGNKENLCKALEYSFADYGDLIKGTSIWDNEYTKDLELNLQNNFGKLFGKYIKKNNTAEQDTSYSSLDELRESWWNTNKKYIWTAMKHGAEMNITTCNADGSVTGSGSSCDDIPTIDLIPQYLRFLQEWVENFCEQRQAKVKDVITNCKSCKESGNKCKTECKTKCKDECEKYKKFIEACGTAGGGIGTAGSPWSKRWDQIYKRYSKHIEDAKRNRKAGTKNCGTSSTTNAAASTDENKCVQSDIDSFFKHLIDIGLTTPSSYLSNVLDDNICGADKAPWTTYTTYTTTEKCNKERDKSKSQSSDTLVVVNVPSPLGNTPYRYKYACQCKIPGGGGSGGGGSAEKKNNKSLCYEKDNDMTWSKKYIKKLENGRSLEGVYVPPRRQQLCLYELFPIIIKNEEGMEKAKEELLETLQIVAEREAYYLWKQYNPTGKGIDDANKKACCAIRGSFYDLEDIIKGNDLVHDEYTKYIDSKLNEIFGSSDTNDIDTKRARTDWWENETITNGTDRKTIRQLVWDAMQSGVRYAVEEKNENFPLCMGVEHIGIAKPQFIRWLEEWTNEFCEKYTKYFEDMKSKCDPPKRADTCGDNSNIECKKACANYTNWLNPKRIEWNGMSNYYNKIYRKSNKESEGGKDYSMIMAPTVIDYLNKRCHGEINGNYICCSCKNIGAYNTTSGTVNKKLQKKETECEEEKGPLDLMNEVLNKMDKKYSAHKM |
| HPISVpmv1 7G8 | MDKSSIGDKIEEYLKEKSDDSKIDELLKADPSEVEYYRSGGDGDYLKNNICKITVNHSDSGKYEPCDRIPPPYGDNDQWKCQQNLYKASENNKNVFVPPRRQRMCINNLENLNVERIRDKHAFLADVLLTARNEGERIVQNHPDTNSSNVCIALERSFADIADIIRGRDGNKCETKSTNNVEELIKKFFEKNYRENKEYKRKYPNDDPKYTKLREDWWTKNRQKVWEVITCGARSNDLLIKRGWTTSKESEGENKLELCRKCGHYEEKVPTKLDYVPQFLRWLTEWIEDLYREKQNLIDDMERHREECTSEDHKSKEGASYCNMCKDKCKKYCECVKKWKSEWENQKNKYKDLYQQENETSSSSKKKSRYDDYVKEFFKKLNEANYSSLENYIKDDPYSKEYVTKLSFIPNSSDANTSSEKIQKNNDEVCNPNESGISSVEQAQTSGPSSNKTCITHSSIKANKKKECKDVKLGVRENDKDLKICVIEDTSLSGVDNCCCQDLLGILQENCSDNKRGSSSNDSCDNKNQDECQKKLDEALESLHNGYKNQKCKSGTSTVNKKWIWKKSSGNKEGLQKEYANTIGLPPRTQSLYLGNLPKLENVSKGVTDIIYDTKEKFLAGCLIVSFHEGKNLKTSHEKKNDDNGKKLCKALEYSFADYGDLIKGTSIWDNEYTKDLELNLQKAFGKLFRKYIKKNISAEQDTSYSSLDELRESWWNTNKKYIWIAMKHGAGMNGTTCCGDGSSGENQTNSCDDIPTIDLIPQYLRFLQEWVEHFCEQRQAKVKDVITNCKSCKNTSGERKIGGTCNGECKTKCKNKCEAYKTFIEHCKGGDGTAGSSWVKRWDQIYKRYSKHIEDAKRNRKAGTKSCGTSTAENKCVQSDIDSFFKHLIDIGLTTPSSYLSIVLDENNCGEDKAPWTTYTTTKNCDIQKDKSKSQSSDTLVVVNVPSPLGNTPHGYKYACQCKIPGGGGSGGGGSAEKKNNKSLCHEKGNDRTWSKKYIKKLENGRTLEGVYVPPRRQQLCLYELFPIIIKNKNDITNAKEELLETLQIVAEREAYYLWKQYNPTGKGIDDANKKACCAIRGSFYDLEDIIKGNDLVHDEYTKYIDSKLNEIFGSSNKNDIETKRARTDWWENETITNGTDRKTIRQLVWDAMQSGVRKAIDEEKEKKKPNENFPPCMGVEHIGIAKPQFIRWLEEWTNEFCEEYTKYFEDMKSNCNRRKGADDCGDNTNIECKKACANYTNWLNPKRIEWNGMSNYYNKIYRKSNKESEDGKDYSMIMEPTVIDYLNKRCNGEINGNYICCSCKNIGAYNTTSGTVNKKLQKKETECEDNKGPLDLMNEVLNKMDEKYSKLNL |
| HPISVpmv1 Mcamp | MDSTSTIANKIEAYLGAKSDDSKIDELLKADPSEVQYYRSGGDGYYLKNNICKITVNHSDSGTNDPCDSIPPPYGDNDQWKCAIILSKVSEKPENVFVPPRRQHMCIKNLEKLNVDKIRDKHAFLADVLLTARKEGERIVQNHPDTNSSNVCNALERSFADIADIIRGTDLWKGTNSNLEKNLKQMFKNILEKGSTIQSNYSKDQNYRKLREDWWNANRQKVWEVITCGARSNDLLIKRGWTTSGKSNGDNKLELCRKCGHYEKEVPTKLDYVPQFLRWLTEWIEDLYREKQNLIDDMERHREECTRKNVDSKEGTSYCSTCKDKCKKYCECVKKWKTEWENQENKYKDLYKKDKRKKSSAKHASRYDDYVKDFFEKLNEANYKSLDDYIKGDPYFAEYATKLSFILNSSDANTSSGETANHNDEACNCNESEISSVEHASISDPSSNKTCNTHSSIKANKKKVCKHVKLGVRENDKDLRVCVIEHTSLSGVENCCFKDFLRILQENCSDNKSGSSSNGSCDKNNEEACEKNLEKVFASLTNCYKCEKCKSEQSKKNNKKWTWRKSSGNKEGLQEEYANTIGLPPRTQSLCLVVCLDEKEGKKTQELKNIRTNSELLKEWIIAAFHEGKNLKPSHEKKNDDNGKKNDDNNSKLCKDLKYSFADYGDLIKGTSIWDNEYTKDLELNLQKIFGKLFRKYIKKNIASDENTLYSSLDELRESWWNTNKKYIWLAMKHGAEMNGTTCNADGSVTGSGSSCDDIPTIDLIPQYLRFLQEWVEHFCKQRQAKVKDVIENCKSCKESGNKCKTECKNKCEAYKKFIENCKGGDGTAGSSWVKRWDQIYMRYSKYIEDAKRNRKAGTKNCGPSSITNVSASTDENKCVQSDIDSFFKHLIDIGLTTPSSYLSIVLDDNICGDDKAPWTTYTTYTTYTTYTTYTTTKNCDKERDKSKSQSCNTAVVVNVPSPLGNTPHEYKYACECRTPGGGGSGGGGSDKGQNNKSLCHEKGNDRTWSKKYIKKLENGRTLEGVYVPPRRQQLCLYELFPIIIKKEKDMEKAKKELLETLQIVAEREAYYLWKQYNPTGKGIDDANKKACCAIRGSFYDLEDIIKGNDLVHDEYTKYIDSKLNKIFGSSNKNDIDTKRARTDWWENETINVANITDTNKSDSKTIRQLVWDAMQSGVRKAIDEEKEKKKPNENFPPCMGVEHIGIAKPQFIRWLEEWTNEFCEKYTKYFGAINSKCNLRKGADDCGDNSNIECKKACANYTNWLNPKRIEWNGMSNYYNKIYRKSNKESEDGKDYSMIMEPTVIDYLNKRCNGEINGNYICCSCKNIGENSTSPVANKKAQRKDEQCEDNKGPLDLMNEVLNKMDEKYSEHKM |
| HPISVpmv1 M200101 | MDSKTTIAEKIEEYLKEKSNDSKIDQSLKADPSEVQYYRSGGDGYYLKNNICKITVNHSDSGTNDPCDRKELSYGDNDQWKCDENLSKVSGKPENICVPPRRQRMCIKNLEKLNVDKIRDKHAFLADVLLTARNEGERIILYHPDTNSSNVCVALERSFADLADIIRGRDGNKCETKSPNNVEELIKKFFEKNYRSNEEYKRKYRNDDENYKKLREAWWNANRQKVWEVITCSARSNDLLIKRRWTTSKESNGENKLELFRKCGHYEKEVPTKLDYVPQFLRWLTEWIADLYREKQNLIDDMERHREECTREDDKSKEVTSYCNMCKDKCKKYCECVKKWKTEWENQKNKYNDLYEQENETSQKNTSRYDDYVKEFFEKLNEANYKSLDDYIKGDPYFAEYATKLSFILNSSDANNPSEKIQKNNDEVCNCNESGISSVEQASISDPSSNKTCNTHSSIKTNKKKECKHVKLGVRENDKVLKICVIEDTSLSGVENCCFKDLLGILQEPRIDKNQSGSSSNGSCDKNSEEICQKKLDEALASLHNGYKCDKCKSGTSTVNKNWIWKKFPGNGEGLQKEYANTIGLPPRTHSLYLGNLPKLENVCEDVKDINFDTKEKFLAGCLIAAFHEGKNLKTTYPQNKKKLCKALKYSFADYGDLIKGTSIWDNDFTKDLELNLQKAFGKLFRKYIKKNIASDENTSYSSLDELRESWWNTNKKYIWLAMKHGAGMNITTCNADGSVTGSGSSCDDIPTIDLIPQYLRFLQEWVEHFCEQRQAKVKDVIKNCNSCKESGGTCNSDCEKKCKIECEKYKKFIEECRTAAEGTAGSSWVKRWDQIYMRYSKYIEDAKRNRKAGTKNCGTSSTTNAAASKCVQSDIDSFFKHLIDIGLTTPSSYLSIVLDENNCGADKAPWTTYTTYTTTKNCDIQKDKSKSQSCDTLVVVNVPSPLGNTPHGYKYACQCKIPGGGGSGGGGSQNNKSLCHEKGNDRTWSKKYIKKLENGRTLEGVYVPPRRQQLCLYELFPIIIKNEEGMEKAKEELLETLQIVAEREAYYLWKQYNPTGKGIDDANKKACCAIRGSFYDLEDIIKGNDLVHDEYTKYIDSKLNEIFGSSNTNNIDTKRARTDWWENETIANGTDPKTIRQLVWDAMQSGVRYAVEKKENFPPCMGVEHIGIAKPQFIRWLEEWTNEFCEKYTKYFEDMKSNCDPPKSADDCDDNSNIECKKACANYANWLNPKRIEWNGMSNYYNKIYRKSNKESEDGKDYSMIMEPTVIDYLNKRCNGEINGNYICCSCKNIGENSTSPAQKTKGSKKKDEQCEDNKGPLDLMNEVLNKMDPKYSKLNL |
