# Supplementary material for: Structure-guided design of VAR2CSA-based immunogens and a cocktail strategy for a placental malaria vaccine
Source: PLoS Pathog. 2024 Mar 4;20(3):e1011879. doi: 10.1371/journal.ppat.1011879 (PMC10939253; doi:10.1371/journal.ppat.1011879)
Supplement: Attachment — Submitted filename: Reviewer Response v6 RM PLOS.docx [file ppat.1011879.s010.docx]

**Response to comments from the Editors:**

***Comment 1.0:*** *Thank you very much for submitting your manuscript "Structure-based design of VAR2CSA and a cocktail strategy for a placental malaria vaccine" for consideration at PLOS Pathogens. As with all papers reviewed by the journal, your manuscript was reviewed by members of the editorial board and by several independent reviewers. In light of the reviews (below this email), we would like to invite the resubmission of a significantly-revised version that takes into account the reviewers' comments.*

We thank the editors for their time and effort in evaluating the reviewer comments and identifying the key areas to address.

***Comment 1.1:*** *1. As suggested by Reviewer 1, more description is needed of the design process and the validation of the immunogens. More information should be provided on how linker lengths were chosen and any other in silico considerations that went into the truncation designs. Additionally, data should be provided that the immunogens folded correctly (secondary structure and protein binding to CSA).*

We thank the reviewer for their time and effort in reviewing the manuscript. As requested by the reviewer, the revised manuscript now contains a comprehensive secondary structural analysis by circular dichroism (CD) (new Figure S3). This analysis reveals all the designed immunogens are correctly folded and resemble the full-length protein. In addition, we have revised figure S1 to depict the correct linkers used and updated the structure-based design section to describe how linkers were defined: “The length and sequence of the flexible linker were determined by evaluating the distance between connecting residues in the structure and designing GS-linker sequences that could accommodate those distances.”

***Comment 1.2:*** *2. The head-to-head serological comparison of full-length VAR2CSA and HPISVpmv1 in figure 3F needs to be titrated below 0.1 mg/ml.*

We agree that additional titrations will strengthen the interpretation that full-length VAR2CSA and HPISVpmv1 produce indistinguishable immune responses. We have updated Figure 3G with an IgG titration range from 0.005-0.5 mg/ml that clearly demonstrates HPISVpmv1 produces as potent blocking inhibitory antibodies as the full-length VAR2CSA.

***Comment 1.3:*** *3. The discussion gives the impression that a cocktail vaccine is a practical approach to achieve strain-transcendence coverage. However, the cocktails did not broaden reactivity beyond the included immunogens. Given that extreme VAR2CSA polymorphism is raised as a major challenge to vaccine development, the authors should acknowledge that the present data suggests these immunogens elicit highly strain-specific inhibitory antibodies and they have not estimated the parasite population diversity of these strain-specific inhibitory epitopes. Therefore, it is difficult to gauge how easy or difficult it will be to design a broad coverage VAR2CSA cocktail. It may be necessary for additional engineering to refocus the antibody response on more shared inhibitory epitopes.*

We must clarify that a large portion of the manuscript (Fig 4-5) is to state that the cocktail study seems only expand the protection to the strains included in the immunization. We also acknowledge that “Future bioinformatic and field surveys will help determine the number of strains to include in this strategy, and clinical trials will be required to determine the efficacy of these combinations.” We have further revised the manuscript in response to these comments:

“The cocktail strategy presented in this study offers a potential approach for a broadly protective placental malaria vaccine and similar practices have been employed for the development of influenza, pneumococcal, and COVID vaccines.”

And:

“Moreover, strain-specific inhibitory epitope mapping may facilitate additional engineering to refocus the antibody response to shared inhibitory epitopes.”

***Comment 1.4:*** *We cannot make any decision about publication until we have seen the revised manuscript and your response to the reviewers' comments. Your revised manuscript is also likely to be sent to reviewers for further evaluation.*

*When you are ready to resubmit, please upload the following:*

*[1] A letter containing a detailed list of your responses to the review comments and a description of the changes you have made in the manuscript. Please note while forming your response, if your article is accepted, you may have the opportunity to make the peer review history publicly available. The record will include editor decision letters (with reviews) and your responses to reviewer comments. If eligible, we will contact you to opt in or out.*

*[2] Two versions of the revised manuscript: one with either highlights or tracked changes denoting where the text has been changed; the other a clean version (uploaded as the manuscript file).*

*Important additional instructions are given below your reviewer comments.*

*Please prepare and submit your revised manuscript within 60 days. If you anticipate any delay, please let us know the expected resubmission date by replying to this email. Please note that revised manuscripts received after the 60-day due date may require evaluation and peer review similar to newly submitted manuscripts.*

*Thank you again for your submission. We hope that our editorial process has been constructive so far, and we welcome your feedback at any time. Please don't hesitate to contact us if you have any questions or comments.*

We thank the editors for their time and effort.

**Response to Reviewer #1:**

***Comment 1.0:*** *The manuscript by Ma et al uses the recent structures of the important pregnancy-associated malaria vaccine candidate VAR2CSA to guide the design of new version of this protein which are more effective as vaccine immunogens. The structures provide detailed insight into the architecture of this complex protein and the location of the ligand binding site, making it timely to take such an approach. Indeed, a complex antigen such as VAR2CSA would benefit from this as it is difficult to make, making a more readily produced antigen desirable.*

We thank the reviewer for their time and effort in reviewing the manuscript and for their positive remarks.

**Comment 1.1:** The approach used is a simple one of just truncating regions of the protein, sometimes replacing domains with linkers. Therefore, while this is “structure-guided”, it does not use the most recent techniques. There is also little data presented to show that these truncations are properly folded, with the authors relying solely on mobility on size exclusion columns and a gel.

We agree with the reviewer that providing evidence in addition to immunological data to demonstrate the immunogens designed retain their expected structures would strengthen the manuscript. In line with comments 1.5 and 1.6 below, we have added a comprehensive analysis of all immunogens by circular dichroism (CD) (new Fig S3). These results establish the designed immunogens retained the expected structures and are similar to the full-length VAR2CSA.

**Comment 1.2:** The authors next test these immunogens by rat immunisation and assessing ELISA signal and the ability of raised sera to block pRBCs from binding to CSA. The main success from the design approach was to generate an immunogen which is produced at higher yield. However, none of the immunogens generated a more effective inhibitory response than full-length VAR2CSA and most (perhaps all?) were less effective. This is interesting as these truncated immunogens were still very large, containing most of the native protein. Why was the quality of the generated antibodies (i.e. the inhibitory activity as a factor of total IgG) not better if only epitopes for non-inhibitory antibodies had been removed?

We thank the reviewer for raising this concern. We would like to emphasize that HPISVpmv1 is approximately 50% the size of the full-length VAR2CSA, expresses over 6-times the yield and produces a potent immune response indistinguishable from the full length VAR2CSA. Women naturally acquire immunity to placental malaria after successive pregnancy and generate antibodies targeting VAR2CSA that block CSA adherence. This indicate that the full-length VAR2CSA is a highly effective immunogen which makes it the leading vaccine candidate for placental malaria. However, it is not an ideal product for industrial manufacturing due to its large size, complex structure, and extensive post-translational modifications (with over 110 cysteines). The goal of this study is to design an immunogen with higher yield and shorter length that retains the critical epitopes and induces inhibitory response equal to full length VAR2CSA. It is also worth mentioning that the shorter length and improved yield of HPISVpmv1 can also reduce the cost of mRNA vaccine production, particularly when multiple variants are required and facilitate nanoparticle vaccine design.

We also must clarify that statistical analysis indicated no significant difference between the antibody titers elicited by HPISVpmv1 and full-length protein (Fig 2b and 3c). Statistical analysis indicated no significant difference between the efficacy of HPISVpmv1 and full-length VAR2CSA by BIA assays with IgGs purified from each individual animal (Fig 3F). Indeed, the additional study requested by this reviewer to further titrate the IgG in the BIA assays further strengthens these inferences.

The question of why the quality of the generated antibodies is not better if only epitopes for non-inhibitory antibodies had been removed is interesting. This is our next-stage study and beyond the scope of this manuscript.

**Comment 1.3:** Finally, the authors show that their immunogen is very variant specific, but also show that immunisation with a ‘cocktail’ of different VAR2CSA variants can lead to a better cross-inhibitory response. This is nice data, although it is not clear that it is related to the ‘structure-based’ design part as the authors do not show whether this only happens with their design or would also occur with full-length VAR2CSA.

This is an interesting scientific question. As stated above, full-length VAR2CSA is not an ideal vaccine for industrial production due to its size and complexity, the goal of this study is to design immunogens with shorter length and improved yield while induce comparable inhibitory activity. This goal was achieved with HPISVpmv1 and therefore HPISVpmv1 was moved forward into the cocktail study.

**Comment 1.4:** In summary, this is a worthwhile study, but there are issues with validation of the designed immunogens and sadly, it appears as though the immunogens generated are not an improvement in terms of immunogenicity, while one does express substantially better. The cocktail approach, which would probably work with full-length VAR2CSA is interesting. There is still work to do to make the best immunogen, as these designs are only a bit shorter than full length are do seem to give slighted lower inhibitory effects.

We thank the reviewer again for the positive remarks and suggestive comments on our manuscript. We must clarify that the size of HPISVpmv1 is only 50% of full-length VAR2CSA, in the meanwhile has a >6 times yield. This benefits industrial production of recombinant vaccine, and also substantially reduce the cost of the production of mRNA-based vaccines, as stated in our manuscript.

**Major Issues:**

**Comment 1.5:** The main concern is the lack of description of the approach used for design and lack of validation of the designed proteins. There is almost no description of the design process. Was it just truncation? When linkers were used, how were they designed? What surfaces on the proteins were exposed on truncation of neighbouring domains and were they fixed or resurfaced?.

We have updated the linker information in Fig S1 and updated the CD analysis to establish that all immunogens are correctly folded. In addition, we have updated the structure-based design section to describe how linkers were defined: “The length and sequence of the flexible linker were determined by evaluating the distance between connecting residues in the structure and designing GS-linker sequences that could accommodate those distances.”

**Comment 1.6:** Also the only demonstration of protein quality is a gel and a SEC trace. Many of the peaks on the SEC trace are very small, making it impossible to see their shape and so at the very least a SEC trace with equivalent loading is essential. In my view, the authors should also present CD traces to show correct secondary structure, SEC-MALLS or the equivalent and demonstration that the proteins bound to CSA with the same affinity as the original. The major reason for concern is that the deltaID1 behaves poorly, while not containing any of the CSA binding residues. Perhaps this has not been designed properly to allow it to fold?

We have now included a comprehensive structural analysis by circular dichroism to establish that all immunogens possess the correct secondary structure. The exact reason why deltaID1 behaves poorly is still unknown. We have included one possible reason in the discussion “a recent study suggests that it may contact DBL2 and DBL4, contributing to the stabilization of the CSA binding channel(44). In addition, although recombinant ID1 alone does not induce a sufficient protective antibody response, it appears indispensable for the induction of adhesion-inhibitory antibodies by either NTS-DBL1 or DBL2(65). Deletion of ID1 described here reduced the stability of multiple distinct designs and their ability to elicit an inhibitory response. This is further evidence that advocates for the inclusion of ID1 as a component of VAR2CSA immunogens.”

**Comment 1.7:** Secondly, sometimes the manuscript does not present a sufficient range of concentrations of sera to determine whether the designed immunogen is as good as the original. For example, Figure 3F only shows concentrations which give very high inhibition and it looks as though the design is slightly worse at the lowest concentration presented than VAR2CSA. Without repeats, stats and measurements at a range of lower concentrations, it is not possible to make a firm conclusion here. An IgG titration is required.

We agree with the reviewer that the original manuscript did not include a sufficiently large titration range. We have updated the manuscript with BIA data for a broader IgG concentration range from 0.005-0.5mg/ml (Fig 3G). These new results are consistent with our original conclusion that HPISVpmv1 produces a blocking inhibitory antibody response equivalent to the full-length VAR2CSA.

Indeed, stats are required throughout as they are missing for most of the data.?

We thank the reviewer for identifying ways to improve the manuscript. We have updated the manuscript with statistical analysis in all figures where appropriate.

**Minor Issues:**

**Comment 1.8:** Through-out the manuscript, the authors use value judgements, with words like “much higher” (line 27), “highly” (line 103), “strong” (line 110), “potent” (line 139) etc etc. In my view, scientific reports should just state the facts in a quantitative way, without using such value judgements.

We thank the reviewer for pointing this out. We have revised the manuscript accordingly.

**Comment 1.9:** I also think that the title is wrong. VAR2CSA is not designed. VAR2CSA-based immunogens?

We thank the reviewer for their valuable suggestion. We have revised the title accordingly.

**Comment 1.10:** Line 37-38 – seems truncated?

We thank the reviewer for pointing this out. We have removed this erroneous sentence.

**Comment 1.11:** Line 59 ‘the design of VAR2CSA’ seems strange phrasing as VAR2CSA is a natural protein. ‘Re-design’, ‘Design based on the structure of VAR2CSA’?

We thank the reviewer for pointing this out. We have revised the manuscript to:

“Structure-guided design of VAR2CSA-based immunogens leveraging the available structural, mechanistic, and immune evasion mechanisms may improve placental malaria vaccines.”

**Comment 1.12:** Figure 3 – please show individual data throughout, rather than just means. Shows states throughout. For F, please titrate down further to IgG concentrations lower than 0.1 mg/ml.

We thank the reviewer for pointing this out. We have updated the figure with statistical analyses where appropriate. Regrettably, it is not appropriate to combine technical replicates of an assay with biological replicates in a figure. We thank the reviewer for identifying how original figure F could be improved and we now present data with an IgG concentrations lower than 0.1 mg/ml. The new data demonstrate that HPISVpmv1 produces a blocking inhibitory antibody response equivalent to the full-length VAR2CSA.

**Comment 1.13-1.16:** Figure 2 – please show individual data throughout, rather than just means. Shows states throughout.

Figure 4 – please show individual data throughout, rather than just means. Shows states throughout.

Figure 5 – please show individual data throughout, rather than just means. Shows states throughout.

Figure S5 + 6 – please show individual data throughout, rather than just means. Shows states throughout.

We thank the reviewer for pointing this out. We have updated the figure with statistical analyses where appropriate. Regrettably, it is not appropriate to combine technical replicates of an assay with biological replicates in a figure.

**Comment 1.17:** Figure S2 – how was loading decided on this set of SEC traces? Some of the curves really can’t be seen at this y axis scale. Show on different scales or repeat with equal loading? What column? How many repeats?

We thank the reviewer for pointing this. We have updated Fig S2 to include each individual SEC trace and state that “The results shown here are one representative purification with all purifications performed at least three times with consistent results.” The methodological information regarding the column and loading for how these traces were obtained were included in the methods section: “The elutes were concentrated with a 100-kDa cutoff centrifugal filter unit (Millipore Sigma) to 1 ml and further purified by size-exclusion chromatography (Superose 6 Increase 10/300, GE Healthcare) in PBS buffer.”

**Comment 1.18:** Figure S3 – I would move this into a main figure as this is the major advantage of the new immunogen.

We thank the reviewer for their valuable suggestion. We have moved Figure S3 to Fig. 3A and revised the text accordingly.

**Response to Reviewer #2:**

**Comment 2.0:** This paper describes the design of various potential vaccine candidates (mainly subunit vaccines) based on knowledge of the VAR2CSA antigens' biological functions and structure . These candidate vaccine antigens were then expressed and used to vaccinate rats. Antibody levels and function were measured. Due to issues in the past of a lack of heterologous immunity being elicited the authors also investigated the possibility of blended or sequential vaccination with antigens from different variants. Antibodies generated using vaccines from multiple variants recognized multiple parasite strains suggesting that a blended vaccine is a plausible approach.
A vaccine against placental malaria would help reduce disease. Hurdles to a vaccine include the large size of the antigen which can make its expression in-vitro difficult as well as a lack of immunity generated to heterologous strains in past sub unit vaccines.
This paper provides potential solutions to both of these queries by showing that subunit vaccines made of antigens from multiple strains can elicit functional antibodies in rats.
This has not been previously shown experimentally and is a significant step forward in the field.
This paper is well written and the methods used are appropriate.

We thank the reviewer for their positive remarks on the manuscript.

Minor Issues

**Comment 2.1:**  Line 63- refer to figure 1B

We thank the reviewer for pointing this out. We have referred figure 1B accordingly.

**Comment 2.2:** Final paragraph in the Introduction- please be clear that this study is in Rats.

We thank the reviewer for pointing this out. We have revised the manuscript accordingly:

-“Serology studies indicated that HPISVpmv1 elicited antibody titers equal to or greater than the full-length VAR2CSA ectodomain control after a two-vaccination regimen in rats.”

**Comment 2.3:** Lines 106- expand abbreviation of the adjuvant CFA/IFA.

We thank the reviewer for pointing this out. We have revised the manuscript accordingly:

“Rats were immunized with the designed immunogens and Complete Freund's Adjuvant (CFA)/Incomplete Freund's Adjuvant (IFA) three times to initially evaluate immunogenicity (Fig. 2A).”

**Comment 2.4:** lines 114-116, consider rephrasing this sentence as it is unclear and incorrect as currently written paragraph starting 140- I needed to reread this multiple times to understand, please rephrase to it is clearer.

We have revised lines 114-116 to read:

“The reduced activity of the ΔID1 versions suggests that elimination of ID1 destabilizes the antigens and is consistent with the reduced expression and yield of the ΔID1 designs.”

***Comment 2.5:*** *line 221- specify which vaccines you are referring to.*

We thank the reviewer for pointing this out. We have revised the manuscript accordingly:

“Second, HPISVpmv1 contains the complete CSA-binding site and inhibitory epitopes on DBL4, which are absent in PRIMVAC and PAMVAC.”

**Comment 2.6:** line 271- specify this is in rats.

We thank the reviewer for pointing this out. We have revised the manuscript accordingly:

“Therefore, in the five-combination group, each HPISVpmv1 variant immunogen induced potent inhibitory activity at a low 2 μg dose in rats.”

**Comment 2.7:** Figure 5- it is really hard to determine what the vaccine was and the antigen was being used in the assays. Please rearrange figure and rewrite figure legend

We thank the reviewer for pointing this out. We apologize the figure is complex, but this is driven by the complexity of the cocktail immunization experiments. We have evaluated alternate figures yet none are simpler than the figure presented. To aid in clarity, we have revised the manuscript to state in the figure legend that: “The cocktail combinations of HPISV strains are shown by the titles of the x-axis”.
